# Audiological Characterization of Individuals with Cornelia de Lange Syndrome *

**DOI:** 10.1055/s-0044-1788001

**Published:** 2024-10-25

**Authors:** Nayara Pereira Santos, Liliane Aparecida Fagundes Silva, Ivone Ferreira Neves-Lobo, Chong Ae Kim, Carla Gentile Matas

**Affiliations:** 1 Department of Physiotherapy, Speech Therapy, and Occupational Therapy, School of Medicine, Universidade de São Paulo (FMUSP), São Paulo, SP, Brazil; 2 Department of Genetics, Instituto da Criança e do Adolescente, Hospital das Clínicas, School of Medicine, Universidade de São Paulo (HCFMUSP), São Paulo, SP, Brazil

**Keywords:** hearing, hearing loss, brainstem auditory evoked potentials, Cornelia de Lange syndrome

## Abstract

**Introduction** Cornelia de Lange Syndrome (CdLS) is a genetic disorder in which individuals may present sensorineural and/or conductive hearing loss, and the results of behavioral auditory assessments are not accurate.

**Objective** To characterize the audiological profile of individuals with CdLS through behavioral, electroacoustic, and electrophysiological audiological assessments.

**Methods** The study included 13 individuals of both sexes, aged between 3 and 26 years, with diagnoses confirmed through genetic studies. The following procedures were performed: medical history survey, otoscopy (pure-tone audiometry [PTA], speech audiometry, and acoustic immittance measures), and auditory brainstem response (ABR).

**Results** In total 62.50% of the participants who underwent PTA had abnormal results (all of which were mild), with a predominance of bilateral conductive hearing loss (60%). Regarding tympanometry, 76.93% had abnormal results, most frequently type B (85.72% on the right and 88.89% on the left ear). Acoustic reflexes showed results compatible with tympanometry changes. Changes in ABR latency values compatible with middle-ear impairment were found in 8 of them (66.66%) – 3 had bilateral (37.50%), and 5 had unilateral impairments (62.50%).

**Conclusion** Mild hearing loss was identified in 62.5% of the individuals with CdLS who underwent the behavioral audiological assessment. In the acoustic immittance measures, 76.9% of the participants presented a tympanometry curve characteristic of middle-ear changes. Acoustic reflexes were absent in 84.6% of the subjects. In the ABR, no changes were identified in auditory pathway integrity. On the other hand, changes in the absolute latency values were found, which are characteristic of conductive hearing loss.

## Introduction

Cornelia de Lange syndrome (CdLS) is a rare genetic polymalformation condition, described by Brachmann in 1916 and by de Lange in 1933, who observed distinct facial traits, upper and/or lower limb abnormalities, intellectual disability, and other associated malformations (cardiac, gastrointestinal, and musculoskeletal) in individuals with the condition. 1 2 3 It has a broad clinical spectrum, ranging from mild phenotypes to severe conditions, which may lead to death. 4

The incidence estimates range from 1:10 thousand to 1:30 thousand live births. The exact incidence rate is unknown because many mild cases tend to be underreported. Most cases result from genetic mutations, equally affecting both sexes, and occurring in all races and ethnicities. 1 4

The diagnosis of CdLS is established in two ways: through clinical findings of the classic phenotypic characteristics of CdLS and/or through the identification of a variant heterozygous pathogen in the *NIPBL* , *RAD2* , *SMC3* , and *BRD4* genes, or a pathogenic homozygous variant in the *HDAC8* or *SMC1A* genes. The rates of these variations are as follows: *NIPBL* – 80%; *SMC1A* – 5%; *HDAC8* –4%; *SMC3* –1 to 2%; and *RAD21* and *BRD4* – < 1%; 5 or by identifying the altered gene, which can be performed through a sequential molecular genetic test of a single gene, be it *NIPBL* , *SMC1A* , *HDAC8* , *SMC3* , *RAD21* or *BRD4* , that is, if the individual has a very characteristic SCdL phenotype and in the initial search variations in the *NIPBL* gene are not found, one should consider of the *SMC1A* , *SMC3* , *RAD21* , *HDAC8* , or *BRD4* genes. All sequences will be searched, one by one, from mild to severe phenotypes. 5

In cases in which it is more difficult to surmise CdLS from observation of the phenotypic features alone, comprehensive genomic testing (which does not require the clinician to determine which genes are likely to be involved) is performed. The technique most widely used is whole exome sequencing (WES), which has been designed to identify and analyze the sequence of all protein-coding genes in the genome. The exome comprises only 1 to 2% of the human genome, but it contains most of the currently-recognized disease-causing variants. 5

As aforementioned, CdLS is a rare syndrome that usually encompasses multiple impairments. Hence, individuals with CdLS may present hearing changes such as outer-, middle-, or inner-ear malformations. Therefore, hearing loss may also be present in various cases, as many genes that can cause genetic hearing loss and be transmitted by autosomal dominant (15%), autosomal recessive (80%), sex-related (2–3%), and mitochondrial patterns (1–2%) have been cataloged. 6 7 8

According to the first international consensus statement, 8 the hearing loss is very common (with rates ranging from 85–90%) in individuals with CdLS, present from childhood, and predominantly bilateral. Conductive hearing loss may be present in 75% of the cases, and sensorineural hearing loss, in 25% of the cases, ranging from mild to severe (40–50%). Sensorineural hearing loss has reported in 45% of adults with CdLS. 8

The etiology of conductive hearing loss in CdLS is usually stenosis of the external auditory canal, middle-ear ossicular anomalies, acute or chronic otitis media, and the presence of nonspecific soft tissue filling the middle ear. The possible causes of sensorineural hearing loss are inner-ear anomalies, such as cochlear dysplasia. 8 9 10

Both conductive and sensorineural hearing losses negatively impact development, particularly that of language, which requires multidisciplinary practices, including routine audiological examinations. 11 Possible surgical (such as ventilation tubes) or nonsurgical treatments must also be considered, as well as the indication, selection, and fitting of hearing aids to potentialize speech and language development, with early intervention in the case of children. As for adults, early intervention aims to improve the interactions among relatives and make communication effective in the workplace, thus maximizing the quality of life of individuals with CdLS. 11

Nonetheless, to date, little is known about audiological profiles in CdLS. 12 Hence, the auditory pathway must be thoroughly investigated, encompassing its peripheral and central portions. Also, since various procedures are used, the most indicated ones must be identified to reach precise diagnoses and guide future studies.

Thus, the objective of the present study was to characterize the audiological profile of individuals with CdLS through behavioral, electroacoustic, and electrophysiological assessments.

## Methods

The present is a cross-sectional observational study addressing the results of audiological assessments of individuals with CdLS, aged 3 to 26 years, with diagnoses confirmed by molecular genetic studies using the WES technique. The research was approved by the institutional Ethics Committee under number 3.317.995.

The following inclusion criteria were applied: individuals of any gender aged between 2 and 45 years, with a medical diagnosis of SCdL of any variant gene, and with a result of the WES confirming the syndrome. The exclusion criterion was individuals who had other syndromes.

The medical records of the patients were analyzed, and 26 cases eligible for the study were selected. However, after contact, one participant died, and twelve patients refused to participate because they lived in other states and could not travel due to the ongoing coronavirus disease 2019 (COVID-19) pandemic and/or due to health conditions. Thus, only 13 (9 male and 4 female) individuals aged between 3 and 26 years were recruited and assessed.

The research began only after the parents/guardians signed an informed consent form and the participants signed an informed assent form. The evaluation was carried out at once, in order to reduce the biases of finding changes due to a possible variation in middle ear conditions.

The following materials and procedures were used:

– The child and adult anamnesis protocol was used to collect the individual's medical history; the guardians of both children and adults were interviewed, given their abnormal intelligence quotient (IQ);
– An Otoscope (Mini 2000, Heine Optotechnik, Gilching, Germany) was used to perform the otoscopy and check for possible obstructions due to the presence of earwax, which would prevent the performance of the audiological procedures;
– An acoustic immittance meter (Madsen Zodiac 901, Natus Medical Incorporated, Middleton, WI, United States) was used to obtain acoustic immittance measurements, and the tympanometry results were classified into curves of types A, B or C. 13 The ipsilateral and contralateral acoustic reflexes were classified as present or absent. 14 If absent, the acoustic reflexes were retested and confirmed;
– An audiometer (Itera II, Natus Medical Incorporated; with supra-aural earphones, model TDH-39, and bone vibrator B-71) was used to perform the pure-tone audiometry (PTA) and speech audiometry, which were conducted at frequencies ranging from 250 to 8,000 Hz. Mean hearing thresholds at 500, 1,000, and 2,000 Hz ≤ 20 dB HL were used in children older than 7 years of age, adolescents, and adults, 15 while mean hearing thresholds at 500, 1,000, and 2,000 Hz ≤ 15 dB were used in children younger than 7 years of age. 16 Hearing loss was classified as either conductive or sensorineural, 17 and the degree of hearing loss was also classified. 15 Speech recognition thresholds (SRTs) and speech recognition percentage indices (SRPIs) were assessed through speech audiometry by having the patients read word lists aloud. 18
– A two-channel equipment (Universal Smart Box Jr TM Smart EP, Intelligent Hearing Systems, North Miami, FL, United States), an abrasive electrolytic paste, and micropore tape were used to perform the auditory brainstem response (ABR) (with ER-3A insert earphones [Etymotic Research, Inc., IL, United States] and/or bone vibrator). This potential was obtained with click stimuli and rarefaction polarity at 80 dBnHL, presented monaurally with ER-3A insert earphones at 27.7 stimuli per second to both children and adults, totaling 2,000 stimuli. Tracing reproducibility was verified; the absolute latencies of waves I, III, and V and interpeak intervals I-III, III-V, and I-V were analyzed at 80 dBnHL, verifying auditory pathway integrity. The results were analyzed qualitatively and quantitatively: in the qualitative analysis, the results were classified as normal when the absolute latencies of waves I, III, and V and interpeak intervals I-III, III-V, and I-V met the normal values proposed in the user manual; 19 in the quantitative analysis, the mean absolute latency values in milliseconds of waves I, III, and V and interpeak intervals I to III, III to V, and I to V were considered.

After collection, the data obtained were tabulated and analyzed, considering the following aspects:

– Pure-tone audiometry: qualitative analysis regarding the presence or absence of hearing loss (altered and normal respectively), as well as the characterization of the type, degree, and laterality of the hearing loss, as well as a quantitative analysis of the auditory thresholds obtained in each frequency;
– Vocal audiometry: qualitative analysis of the normal or altered results in the SRT and word recognition score (WRS), and a quantitative analysis of the thresholds of the SRT and the percentage of correct answers in the WRS;
– Acoustic immittance measurements: qualitative analysis of the presence or absence of alteration and characterization of the type of tympanometric curve, as well as a qualitative analysis of the presence or absence of acoustic reflexes; and
– Auditory brainstem response: qualitative analysis (presence or absence of alteration) and quantitative analysis of the absolute latencies of waves I, III, and V, of interpeaks I to III, III to V, and I to V, and of the electrophysiological threshold.

## Results

### Sample Characterization

Table 1 shows the characterization of the sample in terms of age, gender, and genetic analysis of individuals with SCdL.

**Table 1:** Sample characterization regarding age group, sex, and gene of individuals with Cornelia de Lange syndrome

	Mean	Standard deviation	Minimum	Maximum
*Age (in years)*	12.38	± 7.40	3	26
	Number of participants (N)		Percentage (%)	
*Sex*				
Male	9		69.23%	
Female	4		30.77%	
Total	13		100%	
*Gene*				
NIPBL	12		92.30%	
SMC1A	1		7.70%	
Total	13		100%	

### Pure-Tone Audiometry

Table 2 shows a descriptive analysis of the auditory thresholds obtained in each frequency of the Pure-Tone Audiometry (PTA). No statistically significant differences were found between the right and left ears regarding the mean hearing thresholds at 250 to 8 kHz in the PTA.

**Table 2:** Descriptive analysis of hearing thresholds per frequency obtained with pure-tone threshold audiometry and comparison between right and left ears in individuals with Cornelia de Lange syndrome

	Ear	N	Mean (dB HL)	Standard deviation	Minimum	Quartile 1	Median	Quartile 3	Maximum	W-value	*p* -value ^+^
**0.25 kHz**	RE	8	18.13	9.23	5.00	10.00	20.00	27.50	30.00	7.00	0.2402
	LE	8	20.63	12.66	5.00	6.25	22.50	33.75	35.00		
**0.5 kHz**	RE	8	20.00	15.12	0.00	5.00	22.50	33.75	40.00	0.00	0.0719
	LE	8	22.50	15.81	5.00	5.00	25.00	38.75	40.00		
**1 kHz**	RE	8	21.25	14.33	0.00	6.25	27.50	30.00	40.00	7.50	0.8875
	LE	8	21.25	13.30	5.00	6.25	25.00	32.50	40.00		
**2 kHz**	RE	8	24.38	14.25	5.00	10.00	27.50	38.75	40.00	9.00	0.4299
	LE	8	21.25	16.20	0.00	5.00	25.00	37.50	40.00		
**3 kHz**	RE	8	21.25	14.58	0.00	6.25	25.00	35.00	35.00	4.00	0.4076
	LE	8	19.38	15.68	0.00	1.25	25.00	32.50	40.00		
**4 kHz**	RE	8	21.88	14.87	5.00	5.00	25.00	35.00	40.00	0.00	0.0719
	LE	8	19.38	15.68	0.00	1.25	22.50	35.00	35.00		
**6 kHz**	RE	8	23.75	14.33	5.00	10.00	25.00	38.75	40.00	14.00	0.928
	LE	8	23.75	15.06	5.00	6.25	27.50	38.75	40.00		
**8 kHz**	RE	8	23.75	14.82	5.00	6.25	30.00	35.00	40.00	3.00	0.1198
	LE	8	20.63	12.37	5.00	6.25	25.00	30.00	35.00		

**Abbreviations:** dB HL, decibel – hearing level; LE, left ear; RE, right ear.

**Note:**^+^*p* -value obtained through the Wilcoxon test.

Regarding the type of hearing loss in the PTA, 60% of the patients had bilateral conductive hearing loss, and the other 40% had conductive and sensorineural hearing loss concurrently. As for the distribution per ear in CdLS patients, the PTA found 62.50% of changes in the right ear and 62.5% in the left ear. All hearing losses identified through PTA were mild (N = 5 in the right ear and N = 5 in the left ear).

### Speech Audiometry

In the speech audiometry, which was only performed in eight patients, the SRT and SRPI results were found to be compatible with those of the PTA in all cases.

An inferential analysis was made in these speech tests as well, to compare the right and left ears. However, no statistically significant differences were found (SRT: *p* = 0.6374/ SRPI: *p* = 1.000).

### Acoustic Immittance Measures

The tympanometry results were classified as normal and abnormal per ear (right and left) in individuals with CdLS, with 53.85% presenting abnormal results in the right ear, and 69.24%, in the left ear.

Type-B tympanometry was presented by 85.72% in the right ear and by 88.89% in the left ear, and type-C tympanometry, by 14.28% in the right ear. and by 11.11% in the left ear. There was no association between abnormal results and ear laterality (Pearson Chi-squared test [χ ^2^ ] = 0.650; *p* = 0.420). There was no association between the ears and the type of tympanometry curve change (χ ^2^ = 0.686; *p* = 0.710).

Concerning ipsilateral and contralateral acoustic reflexes, responses were absent in 11 (84.6%) individuals. Only 2 (15.4%) subjects presented bilateral responses.

### Auditory Brainstem Response

Regarding the ABR, Table 3 shows a descriptive analysis of the absolute latency values of waves I, III, and V, and of interpeaks I to III, III to V, and I to V, as well as the electrophysiological threshold.

**Table 3:** Descriptive and inferential analyses of the absolute latency values of waves I, III, and V, interpeak intervals I to III, III to V, and I to V, and ABR Threshold

	Ear	N	Mean	Standard deviation	Minimum	Quartile 1	Median	Quartile 3	Maximum	W-value	*p* -value ^+^
**Wave I**	RE	12	1.74	0.36	1.43	1.52	1.64	1.85	2.75	16.00	0.275
	LE	12	1.84	0.31	1.45	1.63	1.72	2.15	2.40		
**Wave III**	RE	12	3.85	0.25	3.69	3.70	3.78	3.87	4.60	16.50	0.084
	LE	12	4.01	0.35	3.62	3.71	3.94	4.34	4.70		
**Wave V**	RE	12	5.67	0.25	5.45	5.55	5.60	5.68	6.40	12.00	0.034*
	LE	12	5.84	0.33	5.45	5.54	5.84	6.07	6.57		
**Interpeak interval I–III**	RE	12	2.12	0.18	1.83	1.95	2.18	2.27	2.35	24.50	0.476
	LE	12	2.16	0.14	1.95	2.04	2.14	2.29	2.40		
**Interpeak interval III–V**	RE	12	1.82	0.07	1.70	1.79	1.81	1.85	1.97	30.00	0.823
	LE	12	1.83	0.19	1.52	1.63	1.89	1.97	2.12		
**Interpeak interval I–V**	RE	12	3.94	0.17	3.65	3.85	3.94	4.08	4.18	30.50	0.529
	LE	12	3.99	0.14	3.80	3.87	4.00	4.15	4.18		
**ABR threshold**	RE LE	12 12	36.67 39.58	14.35 16.02	20.00 20.00	20.00 22.50	40.00 40.00	50.00 50.00	60.00 70.00	2.50	0.203

Abbreviations: ABR, auditory brainstem response; LE, left ear; RE, right ear. Notes: *Statistically significant difference; ^+^ *p* -value obtained through the Wilcoxon test.

The comparison of the results of the right and left ears through the Wilcoxon test revealed a difference between them only in the absolute latency of wave V in the ABR. The left ear presented higher values than those of the right ear ( *p* = 0.034).

Table 4 shows the qualitative analysis of the ABRs, which were classified as normal or abnormal in the right and left ears of individuals with SCdL.

**Table 4:** Qualitative analysis of the ABR (normal or abnormal) and *p* -value of the association between the right and left ears in individuals with Cornelia de Lange syndrome (N = 12)

	Ear	Normal	Abnormal	Chi-squared	*p* -value ^+^
**Wave I**	RE	75.00%	25.00%	1.600	0.206
	LE	50.00%	50.00%		
**Wave III**	RE	83.33%	16.67%	1.815	0.178
	LE	50.00%	50.00%		
**Wave V**	RE	91.67%	8.33%	2.459	0.104
	LE	50.00%	50.00%		
**Interpeak interval I–III**	RE	100.00%	0.00%	Ө	Ө
	LE	100.00%	0.00%		
**Interpeak interval III–V**	RE	100.00%	0.00%	Ө	Ө
	LE	91.67%	8.33%		
**Interpeak interval I–V**	RE	100.00%	0.00%	Ө	Ө
	LE	100.00%	0.00%		
**ABR threshold**	RE LE	33.33% 25.00%	66.67% 75.00%	**0.202**	**0.653**

**Abbreviations:** ABR, auditory brainstem response; LE, left ear; RE, right ear.

**Notes:** Ө: the analysis could not be performed because the samples were too similar; ^+^ *p* -value obtained through the Pearson Chi-squared test.

No statistically significant difference was found regarding the changes in the absolute latencies of waves I, III, and V and interpeak intervals I-III, III-V, and I-V, and the ABR threshold between right and left ears.

## Discussion

In the present study, we assessed 13 individuals with CdLS – 12 with a genetic diagnosis of *NIPBL* variant (92.30%), and 1 with a diagnosis of *SMC1A* variant (7.70%), as shown in Table 1 .

The PTA could only be performed in 8 out of the 13 individuals, and hearing loss was detected in 5 of them (62.50%), as shown in Tables 2 and 3 . Some studies have found a an incidence of hearing loss ranging from 60 to 67% in subjects assessed through PTA, 9 20 whereas other similar studies found that hearing loss affected 80% of the individuals with CdLS. 5 10 11 21 This demonstrates an unequal occurrence of hearing loss in the population in question. Many factors, such as the gene variant and the level of organic or cognitive impairment, are believed to interfere with this divergence.

As for the type, there were 3 cases of bilateral conductive hearing loss (60% of the patients with hearing loss) and 2 cases of right-ear sensorineural and left-ear conductive hearing loss (40% of the cases of hearing loss). Thus, all individuals presented conductive hearing loss, most of them bilaterally. Likewise, studies 11 21 have found conductive hearing loss in 60% and 59.1% of the cases. Elaborating on the audiological findings, genetic diagnoses, and clinical severity of the individuals with CdLS, some studies 21 highlighted the association with conductive hearing loss in a considerable number of individuals with more severe phenotypes ( *NIPBL* variant), corroborating the findings of the present study, in which this variant and type of hearing loss were predominant. This can be explained by the findings of middle-ear impairments in individuals with CdLS, particularly due to soft-tissue malformations or fluid present in the middle-ear cavity. 9 10 Some authors 21 have stated that middle-ear impairments in individuals with CdLS may be associated with *NIPBL* mutations, as no *NIPBL* mutation was found in normal hearing individuals – although they pointed out that further studies assessing new variants identified in CdLS are needed to confirm this finding.

Some treatment options, such as drugs and/or ventilation tubes, can be approached to minimize the negative impacts of conductive hearing loss. However, they are not always reliable clinical resources for these individuals because some of them have middle-ear malformations (soft-tissue lesions) – rather than fluid effusion, which usually takes place in secretory otitis media –, verified through imaging examinations and in intraoperative conditions. 10 Hence, the possible conductive hearing loss etiologies in this population include stenosis of the external auditory canal, middle-ear ossicular anomalies, nonspecific middle-ear anomalies (nonspecific soft tissues filling the middle ear), and acute or chronic otitis media. 12

Sensorineural hearing loss corresponded to 40% of the types verified in the present study, all of them unilateral and coexisting with contralateral conductive hearing loss. This corroborates to some extent the findings of another study, 12 which verified sensorineural hearing loss in 40.3% of the cases. However, the authors 12 reported that sensorineural hearing loss was the most common impairment in CdLS, followed by conductive hearing loss – which does not agree with the data obtained in the present research. 12 Neither was sensorineural hearing loss the most frequent type in other studies, corresponding to 22.7% 21 and 33.3% 20 of the cases, slightly below what was found in the present study. According to some authors, 12 the possible sensorineural hearing loss etiologies in this population include inner-ear malformations, such as cochlear dysplasia.

Regarding the degree, the present study verified bilaterally mild hearing loss through PTA in all cases, corroborating the findings of other studies 9 11 21 22 23 in which mild hearing loss also predominated. However, other authors 20 24 25 found moderately severe hearing loss as the most frequent one, while another study 26 verified profound hearing loss to be prevalent (about 90% of the cases).

Such relevant variability in the degree of hearing loss may be explained when considering that CdLS is rather heterogeneous and that, though individuals may have the same genetic diagnosis, their phenotypic expressions are complex and diversified. It is known that the more severe the phenotypic expression in an individual with CdLS, the more systems and/or organs may be affected – and auditory structures are more likely to be impaired in such cases. Hence, the greater occurrence of mild hearing loss may be explained by the individuals' milder phenotypes and cognitive conditions to understand and respond to what they were asked in the PTA, suggesting that their changes in auditory structure were likewise milder.

Speech test results in speech audiometry were compatible with PTA results, indicating that the audiometry was reliably conducted. The SRPI did not suggest retrocochlear impairments, confirming the other findings of predominant middle-ear changes.

Tympanometry was conducted in all 13 study patients. Of these, 53.85% presented changes in the right ear, and 69.24%, in the left ear. The main change found was the type-B tympanometry curve (85.72% of right-ear and 88.89% of left-ear changes), followed by the type-C curve (14.28% of right-ear and 11.11% of left-ear changes), which, in 2 individuals, were only found in 1 ear.

Few studies have described the results of acoustic immittance measures. One of them 10 took such measures from 14 individuals with CdLS, finding type-B tympanometry curves in 13 of them (92.85%).

The occurrence of type-B tympanometry curves in both the present and other studies 10 11 21 23 27 corroborates the type of hearing loss most frequently found in this population (conductive), as previously reported.

The Acoustic reflex results were compatible with the changes found in the tympanometry. Only two of the studies 22 27 that verified acoustic immittance measures reported data on acoustic reflexes: one 22 described bilaterally present acoustic reflexes, corroborating mild sensorineural hearing loss, and the other 27 described a case of bilaterally-absent reflexes due to middle-ear impairment (type-B tympanometry curve). 27

In CdLS, middle-ear impairment commonly results from malformations and/or nonspecific tissues in the cavity, not necessarily from fluids present in the middle ear. Further studies with complementary imaging diagnoses are also needed to identify possible cases of treatable changes (otitis media caused by fluids present in the cavity).

The ABR results, regardless of the parameters analyzed, revealed abnormal latency values in 8 (66.66%) of the 12 individuals assessed. Bilateral change was observed in 3 (37.50%) of them, and 5 presented unilateral change (62.50%).

The following changes were found in ABR: increased absolute latency in wave I (25% in the right ear and 50% in the left ear), in wave III (16.67% in the right ear and 50% in the left ear), and in wave V (8.33% in the right ear and 50% in the left ear). Changes in the interpeak interval III to V were only found in one case and only in the left ear, whereas no changes in interpeak intervals I-III and I-V were found in any of the cases ( Table 4 ).

The change observed in interpeak interval III to V was a slight increase. However, this is an isolated finding, as the interpeak interval I to V was normal. Thus, it was not qualified as suggestive of changes in the upper brainstem.

Few studies 27 28 have described brainstem auditory pathway integrity analysis in individuals with CdLS, as most of them only reported electrophysiological threshold findings.

In one of the studies, 28 the authors investigated auditory pathway integrity through ABR in two individuals with CdLS. One of them presented normal latency in wave I and interpeak interval I to V at 80 dBnHL, while, in the other one, responses were absent at 100 dBnHL. Since they did not find any external acoustic meatus or tympanic membrane abnormalities, the authors 28 concluded that the first case was of mild sensorineural hearing loss, and the second, of profound sensorineural hearing loss; however, other audiological procedures would be necessary to confirm these findings.

In a case study, 27 the authors found increased absolute latencies in waves I, III, and V, and normal interpeak intervals, bilaterally, in the ABR results. In the acoustic immittance measures, they found type-B tympanometry curve and absent acoustic reflexes, bilaterally, confirming the conductive hearing loss. 27 Hence, further studies with ABR in this population are needed to investigate possible impairments in brainstem auditory pathway integrity – which, in combination with other tests, may help reach a better audiological diagnosis.

Table 3 shows a statistically significant difference between the right and left ears only for the absolute latency of wave V – with higher values in the left ear. This may be due to the more tympanometry curve changes (type B) in the left ear, characterizing middle-ear changes and causing absolute latency delay in ABR.

As aforementioned, ABR changes in individuals with CdLS may be due to middle-ear impairments. In the present study, one individual had increased absolute latencies in waves I, III, and V, and normal interpeak intervals, bilaterally, while four individuals had the same condition unilaterally – all of them with tympanometry curves of types B and C.

Moreover, two individuals had decreased latency of wave I, with a tympanometry type-A curve. This finding could be hypothetically compared with studies in individuals with Down syndrome (DS) who have decreased latency values of waves I, III, and V in the ABR, either having or not hearing loss. 29

There is no consensus in the literature about what causes precocious ABR latency values in DS. One of the various hypotheses is that the smaller head circumference decreases the distance between the cochlea and the brainstem. 30 Therefore, a similar situation can be supposed in individuals with CdLS, as they have some craniofacial traits, including microcephalia, like those of individuals with DS. 5 Studies 30 in individuals with DS also suggest possible early brainstem myelination, auditory pathway change or simplification, and/or greater nervous fiber conduction speed and smaller brainstem – which can apply to some cases of CdLS as well.

The present study also verified that four individuals presented perfectly normal absolute latencies of waves I, III, and V, and interpeak intervals, two of them, bilateral, and two, unilateral, but with a type-B tympanometric curve. Hence, it can be deduced that middle-ear impairments delayed the absolute latency of the waves, but, since they are naturally precocious, the conditions in certain cases of CdLS may have led to normal latency values. Hence, middle-ear conditions must be investigated to adequately interpret ABR normal latency values in individuals with CdLS.

There is not satisfatory investigation about the peripheral and central auditory pathway in individuals with SCdL. The full evaluation of the auditory system is a differencial for this study.

Most studies 12 on SCdL found hearing loss in this population of patients, predominantly conductive hearing loss, followed by sensorineural hearing loss, which corroborates the results of the present study, in which conductive hearing loss was the most frequent finding. This finding could be confirmed with the use of different procedures to compose the audiological evaluation battery, since certain procedures cannot be performed in the population in question, usually due to the intellectual impairment they present.

Still, mild hearing loss was predominantly found in the present study, corroborating the findings of other studies, 9 11 21 22 23 which indicate that approximately one third of the individuals present mild hearing loss.

Regarding the establishment of more advisable audiological procedures for this population, we emphasize that, in cases of more severe phenotypic expressiveness, these patients may benefit from objective evaluation methods, mainly acoustic immittance measures to verify middle-ear conditions, and ABR electrophysiological threshold to verify the degree of auditory impairment, since PTA is not always feasible due to behavioral and IQ alterations.

Also, as aforementioned, SCdL is a rare genetic disease, with little-known and previously researched conditions. Most of the research 9 22 23 24 25 26 27 28 has relied on case studies, and, as SCdL presents heterogeneity within the population itself (although *NIPBL* individuals present variation in the same gene, for example, they may present different phenotypes, from milder to severe), it is difficult to find similar data to group the patients.

Thus, hearing loss in individuals with SCdL should be a concern and not be accepted merely as an alteration inherent to the syndrome. Therefore, the need for early assessment, otorhinolaryngological and audiological follow-up, and intervention in individuals with SCdL is highlighted, from routine audiological examinations to strategies to treat hearing loss.

Early intervention will enable the development of language and auditory skills, providing a better quality of life for individuals with SCdL and their families in all aspects: verbal, cognitive, social and professional.

To rule out the impairments that hearing loss can cause in individuals with SCdL, it is important to develop public policy guidelines on the subject. Therefore, there must be a wide range of diagnostic tests: PTA, ABR and acoustic immittance measures and, if any alteration is detected, these individuals can be referred for the use of: hearing aid device (HAD), bone-anchored hearing aid (BAHA), and cochlear implant (CI), because, according to research, 12 some degree of success has been obtained when using these devices.

The limitations of the present study include the small sample size, as it is a rare syndrome. Additionally, many individuals invited to participate lived in other states, making their attendance difficult; adherence to the study was further impacted by the COVID-19 pandemic. Moreover, some individuals had difficulties performing all procedures because of specific issues of the syndrome, such as IQ and behavioral changes.

## Conclusion

The behavioral, electroacoustic, and electrophysiological hearing assessments in individuals with CdLS in the present study have revealed that:

- In the PTA, 62.5% of the individuals with CdLS who could undergo the test presented hearing loss.
- Mild conductive hearing loss was the most frequently found type and degree.
- Acoustic immittance measures identified 76.93% of individuals with middle-ear impairments. Acoustic reflexes were compatible with tympanometry injuries.
- Increased ABR latencies were found in 61.5% of the individuals, which is compatible with middle–ear impairments.
- In some cases, the absolute latency values were found to be normal, even with abnormal tympanometry results – which suggests that ABR results should be always analyzed in combination with the other procedures.
